# Acute Calcific Cerebral Embolism Large Vessel Occlusion: A Unique Stroke Mechanism With Hard Challenges

**DOI:** 10.7759/cureus.22605

**Published:** 2022-02-25

**Authors:** Rea Mittal, Yael Pinero Colon, Ephraim W Church, Anil Yallapragada

**Affiliations:** 1 Neurology, Penn State College of Medicine, Hershey, USA; 2 Neurology, Penn State Health Milton S. Hershey Medical Center, Hershey, USA; 3 Neurosurgery, Penn State Health Milton S. Hershey Medical Center, Hershey, USA

**Keywords:** computer tomography, tissue plasminogen activator, ischemic stroke, thromboembolic stroke, stroke characteristics, stroke outcome, stroke intervention, rare cause of stroke, stroke complications, stroke guidelines

## Abstract

We present the case of an ischemic stroke associated with partially occlusive acute calcified cerebral emboli large vessel occlusion (CCE LVO). No revascularization strategy guidelines have been established for this unique acute ischemic stroke population, although many studies have reported impaired and inconsistent responses to both thrombolysis and thrombectomy. The patient in this case report, unfortunately, experienced a failed attempt at complete thrombolysis, resulting in a poor clinical outcome. Endovascular thrombectomy was not performed because of incomplete obstruction and risk of injury. Follow-up imaging revealed an acute ischemic stroke at the large middle cerebral artery and a new intraparenchymal hemorrhage with complete absence of the previously identified calcified embolus. This case and current literature demonstrate that more data are needed to determine the best revascularization approach for patients with CCE LVO stroke. With tissue plasminogen activator marginally effective in these patients, thrombectomy should be considered in highly unstable, clinically symptomatic patients even only with partial vessel occlusion.

## Introduction

Calcified cerebral emboli (CCE) are a rare cause of acute ischemic stroke, with an estimated incidence of 2.7%-5.9% [1,2]. Intravenous (IV) tissue plasminogen activator (tPA) and/or endovascular thrombectomy (ET) for the treatment of calcified cerebral emboli large vessel occlusions (CCE LVO) has been reportedly associated with unique challenges with poor outcomes. This might be attributable to the unique characteristics of these types of occlusions, specifically tPA resistance due to lack of fibrin content [1,3] and increased vascular injury during ET from hard and irregular CCE properties [4]. We describe the case of a patient with left M1 calcific emboli that were underappreciated on the initial non-contrast computed tomography of the head (NCCTH); the patient received tPA alone and subsequently developed ischemic stroke with the hemorrhagic transformation of a large middle cerebral artery (MCA) territory with an unfavorable clinical outcome.

## Case presentation

A 77-year-old Caucasian woman with a medical history of hypertension, hyperlipidemia, type 2 diabetes mellitus, and status post-transcatheter aortic valve procedure one year before presentation, coronary artery disease, and paroxysmal atrial fibrillation not treated with anticoagulation developed acute-onset left MCA syndrome 90 minutes before hospital arrival. Her NIH Stroke Scale score was 28, most significant for global aphasia, left gaze preference, and right hemiparesis.

**Figure 1:**
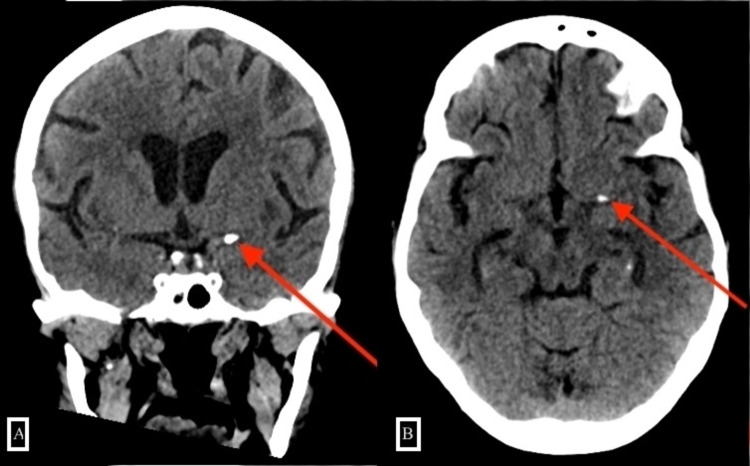
Nonenhanced CT of the head shows a calcified embolus within the left M1 segment of the MCA. MCA, middle cerebral artery

Her initial NCCTH demonstrated an Alberta Stroke Program Early computed tomography (CT) score of 10 and increased hyperdensity in the left M1 segment of the MCA consistent with a “hyperdense MCA sign” (Figure 1).

**Figure 2:**
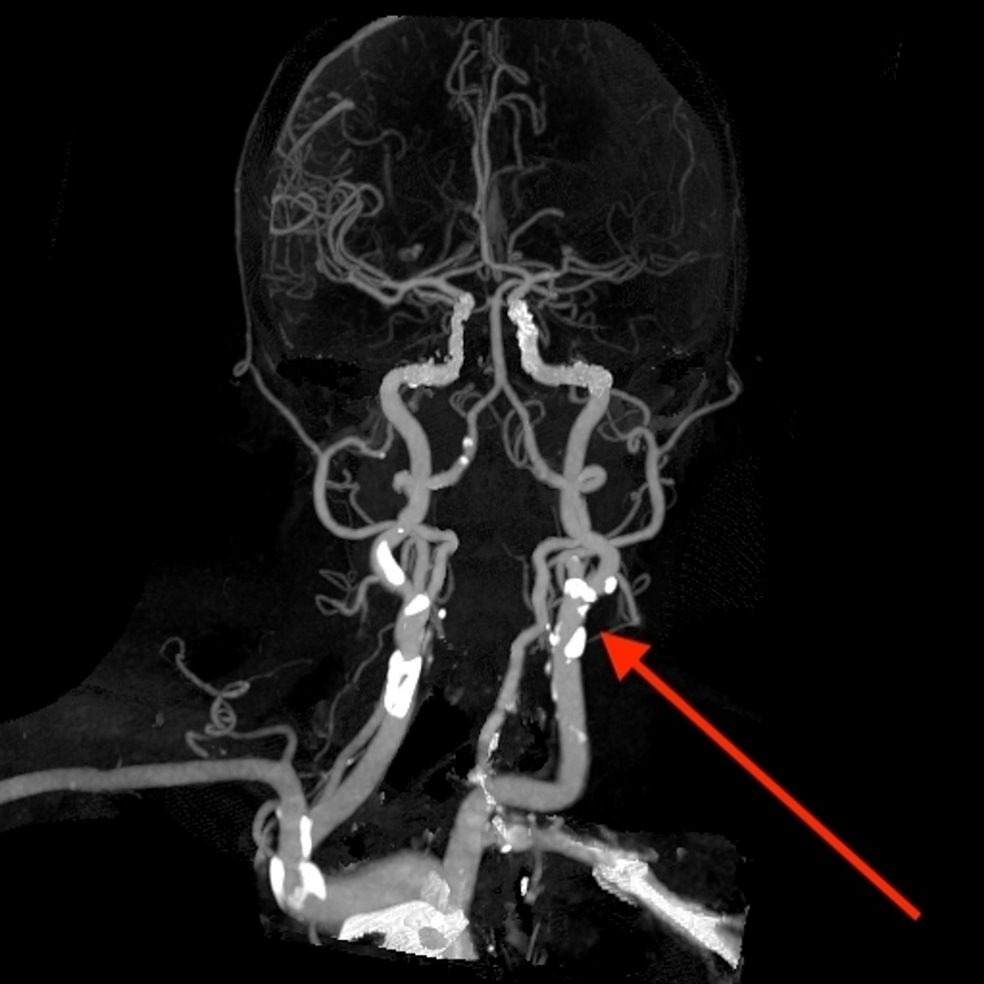
CT angiogram of the head and neck shows showing irregular calcified atherosclerotic plaques.

CT angiogram showed a left M1 calcified filling defect diagnosed as CCE LVO. A heavily calcified atheroma was also detected within the aortic arch, left common carotid, and cavernous segment of the left internal carotid, which were identified as probable sources of emboli (Figure 2).

CT perfusion was not performed at the early presentation.

**Figure 3:**
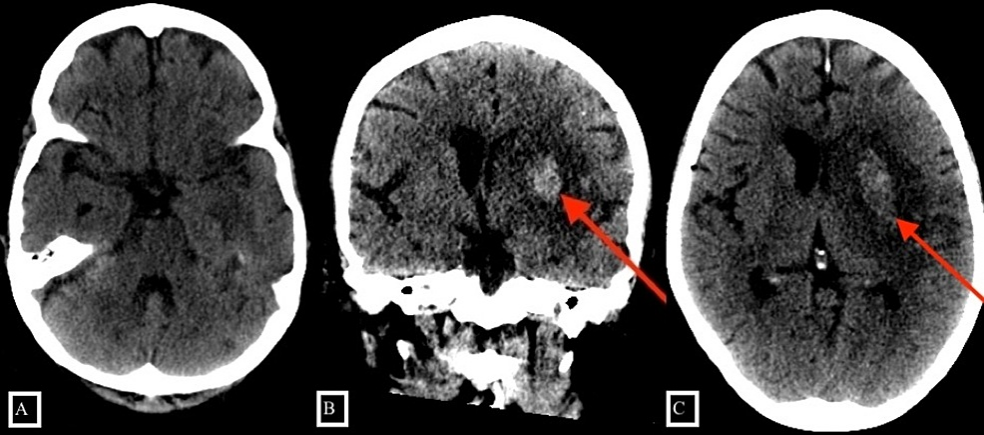
A 24-hour post tPA CT of the head shows no evidence of calcified emboli in the left M1 segment with intraparenchymal hemorrhage in the left putamen. tPA, tissue plasminogen activator

The patient was administered IV tPA within 30 minutes of arrival and admitted to the neurology intensive care unit. A 24-hour post-tPA NCCTH revealed an acute ischemic stroke at the large MCA territory with a 3.4-cm intraparenchymal hemorrhage in the left putamen and complete absence of the previously appreciated CCE LVO (Figure 3).

Follow-up brain MRI confirmed a large ischemic injury in >50% of the left MCA territory with a 3-mm rightward midline shift. Her clinical condition worsened, and thus she was transitioned to hospice care.

## Discussion

CCE ischemic strokes rarely occur, with <200 cases reported in the literature [5]. Walker et al. found that 27% of the identified cases were misdiagnosed using NCCTH and 9% were overlooked on initial interpretation. Calcific embolic sources have been reported to spontaneously originate from cardiac, aortic, and carotid disease, or following manual dislodgement after an invasive procedure [5]. Most calcified emboli lodge in the MCA and are characterized by round- or ovoid-shaped imaging findings and increased attenuation (162 HU) when compared to an intraluminal thrombus (50-70 HU) [5].

Here, we describe a case of CCE LVO in the left M1 segment treated with IV tPA. The patient did not have clinical improvement, and follow-up imaging demonstrated hemorrhagic transformation with a large ischemic territory. ET was not considered as an initial option because the CCE LVO was partially occlusive with the distal filling of the vessel on the CTA.

Unfortunately, CCE LVO is a unique ischemic stroke subtype without an established best approach for recanalization. Data comparing the efficacy of acute thrombolytic therapy as a treatment of calcific and non-calcific embolic strokes are limited [6]. The efficacy of thrombolysis with tPA increases with the proportion of red blood cells within the thromboembolic material and decreases with its fibrin content. Therefore, the dissolution of calcified embolus by tPA is challenged due to its predominant calcium and cholesterol compositions, unless it is associated with fibrin-rich thrombus [7].

ET has been suggested as an alternative therapy, although no definite treatment guidelines have been established. One retrospective multicenter study of 40 patients with CCE who underwent ET reported modified thrombolysis in cerebral infarction score of >2b in 57.5% of patients and excellent outcome (mRS 0-1) in 20.6% of patients [2]. Co-administration of tPA did not lead to better outcomes or more complications [2]. However, patients who underwent ET may have worse angiographic outcomes and a higher mortality rate when compared with noncalcified thrombi. A lower recanalization rate during ET may be caused by stent retrievers designed to integrate the thrombus into the stent cells and disintegration of CCE composition and other thrombus types. The distinct friction properties of hard calcified clots may also adhere lesser to the stent retriever. Similarly, Bruggeman et al. found that 44% of 50 patients with CCE successfully received ET reperfusion. Patients who were co-administered tPA did not have different reperfusion or functional outcomes than those not receiving tPA [8].

Ischemia-reperfusion injuries are common after revascularization therapy and more common in patients with CCE than those without. Bruggeman et al. reported that symptomatic intracranial hemorrhage at post-treatment was observed in 15% of patients and 6% of patients without CCE [8]. Although recanalization is beneficial, delayed recanalization and reperfusion in the settings of low or mixed fibrin-based emboli, such as CCE LVO, can induce free radical damage, neuroinflammation, and neuron apoptosis, resulting in complications such as edema, hemorrhage, and midline shift [7]. Calcific emboli that progressed into distal migration after tPA administration have also been reported [9]. These findings demonstrate that although some calcific emboli may lyse with tPA, such patients should be closely monitored for distal or recurrent embolization [9].

In summary, an impaired response of CCE LVO to tPA may suggest delayed or ineffective recanalization with subsequent increased risk of reperfusion injury, hemorrhagic transformation, or persistent occlusion. Consistent with the current literature, we hypothesize that immediate ET should be considered in select patients with CCE LVO who exhibit evidence of poor collaterals and impaired perfusion in both partial and complete occlusions, a highly unstable acute ischemic stroke population. A direct aspiration approach using large-bore aspiration catheters may improve the outcomes [10]. Further studies are needed to better guide practitioners in selecting patients for the optimal revascularization strategy in CCE LVO.

## Conclusions

Acutely symptomatic patients with a high-grade acute partial or complete CCE LVO should be considered critically unstable and may not be responsive to isolated thrombolytic therapy. Therefore, immediate ET should be considered in select patients with CCE LVO who exhibit a debilitating neurological presentation, evidence of poor collaterals, and impaired perfusion. A direct aspiration approach may also be useful.
